# Numerical Analysis of Standing Waves Phenomenon of Aircraft Tires

**DOI:** 10.3390/ma15092960

**Published:** 2022-04-19

**Authors:** Yu Gan, Xingbo Fang, Xiaohui Wei

**Affiliations:** 1 Key Laboratory of Fundamental Science for National Defense-Advanced Design Technology of Flight Vehicle, Nanjing University of Aeronautics & Astronautics, Nanjing 210016, China; gan_yu@nuaa.edu.cn (Y.G.); fangxingbo@nuaa.edu.cn (X.F.); 2 State Key Laboratory of Mechanics and Control of Mechanical Structures, Nanjing University of Aeronautics and Astronautics, Nanjing 210016, China

**Keywords:** aircraft tires, finite element method, standing waves

## Abstract

In this paper, the generation mechanisms of standing waves on aircraft tires are discussed by comparing the time-domain model and FEA model. Unlike passenger car tires, aircraft tires accelerate to landing speed sharply when the airplane lands. According to the tire structure, the detailed finite element model of the aircraft tires is established in ABAQUS. The tire model runs on a 1.7 m spinning drum and accelerates to 300 km/h in 0.3 s. The proposed finite element model is verified by comparing the simulation results under inflation and static load with the experimental results. Similarly, by analyzing and comparing the calculated values of the time-domain model and FEA model, the variation of standing wave wavelength at different speeds is studied. In addition, the stress and strain field of the aircraft tires standing wave is analyzed. According to the definition of tire standing wave, a method for determining critical speed is proposed. Finally, the effects of tire inflation pressure and vertical load on the occurrence of standing waves were studied.

## 1. Introduction

In recent years, aircrafts’ increased size and operating speeds, especially hypersonic flight vehicles, have required higher takeoff and landing speed. The trend in the design of future aircraft and aerospace vehicles is toward higher speed requirements. When rolling at high speed, waves or ripples are formed on a tire behind the area of contact with the road or runway. In practice, the effect is most significant on the tires of aircraft with high landing and takeoff speeds (300–400 km/h) and high loadings. The repeated deformations caused by the wave process result in considerable heat build-up, causing strength losses in the tire and its total disintegration if sustained. Because these waves appear stationary to the observer, they become known as standing or stationary waves. The current research is mostly focused on automobile tires; the preceding requirements are significantly beyond existing technology, so it is necessary to study the standing wave characteristics of aircraft tires.

Many standing waves studies on automobile tires have been performed, whereas only a few on aircraft tires exist. As described in Ames’s paper [1], many theoretical models of wave phenomenon had been published before 1970. At present, the finite element method has been widely used in tire mechanics analysis, which improves the ability to design a tire structure.

In the study of standing wave theory, Padovan [2] studies the effect of structural damping on the development of standing wave phenomena based on the classic tire base ring model. Brockman et al. [3] introduced a semi-analytical method for predicting standing wave motions (critical speed phenomena) in pneumatic tires. Cusumano and Zolock [4] designed a small test rig for testing tire standing waves and calculated the spatial frequency of standing waves under different pressures, respectively. Chatterjee et al. [5,6] verified experiments on small-diameter tires through a single-degree-of-freedom toroidal membrane model. Based on the analysis, a method for suppressing the standing waves was proposed and confirmed experimentally. Padovan et al. [7] proposed a method that can deal with the standing wave response of aircraft tires and described the rolling tire system as a large deformation nonlinear eigenvalue problem.

In the research of finite element modeling, Chang et al. [8] studied the dynamic response characteristic of tire standing waves and established a finite element model of car tires/drums. Cho et al. [9] analyzed the standing wave dynamic characteristics of three-dimensional tread tire models by comparing the test results and the FEA model results. Sun et al. [10] analyzed the tire load-deflection relation by comparing the FEA results and the experimental results and studied the changes of sidewall standing wave wavelength at different speeds. Kakavas [11] established the mixed finite element formula for large deformation near the incompressible elastic butt joint. Masoudi [12] studied the three-dimensional fatigue crack growth failure model and fatigue life prediction of high chromium steel roll during hot rolling in order to minimize the roll failure through practical solutions. Masoudi [13] calculates stress intensity factors numerically using the boundary element method in railway wheels based on experimental data of material behavior. Nejad [14] predicts rolling contact fatigue crack propagation of wheels under mechanical load and stress field during heat treatment.

It is worth noting that few studies have been published on standing waves of an aircraft tire. This paper mainly analyzes the generation mechanisms of standing waves on the aircraft tire with high landing and takeoff speeds and high loadings by comparing the time-domain model and the FEA model. The finite element model of aircraft tire standing wave dynamic characteristics is established in ABAQUS (Dassault Systems SIMULIA, Providence, Rhode Island, USA). The simulation results under inflation and static load are compared with the experimental results to verify the effectiveness of the model. The effects of tire inflation pressure and vertical load on standing wave dynamic problems are studied parametrically. This study proposes a method for judging the critical velocity of standing waves, which provides an effective reference for the design of aircraft tires.

## 2. Standing Wave Theory

**Figure 1:**
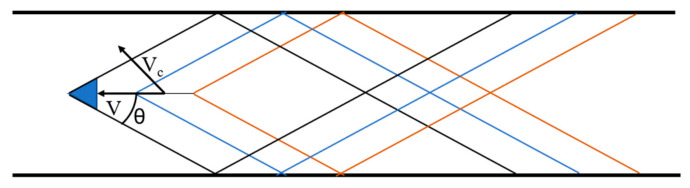
The wave pattern produced by the object traveling in a channel.

Assuming that the standing wave is explained by the object wave in the channel, the object in the channel moves faster than the wave propagation speed, as shown in Figure 1. The angle of the wave θ is expressed as

(1) $$\sin\theta =V_{c}/V$$

Among them V is the object speed and $V_{c}$ is the propagation speed of the wave. Because the moving speed of the diamond wave is the same as that of the object, it looks stationary when viewed from the object. This is similar to when the tire expands to the flat plate. The external force corresponds to the object in Figure 1. When the tire speed is higher than the wave speed in the tire, the standing wave occurs.

Thanks to the powerful processing power of the computer, a time-domain model was developed to study the standing wave in the tire.

**Figure 2:**
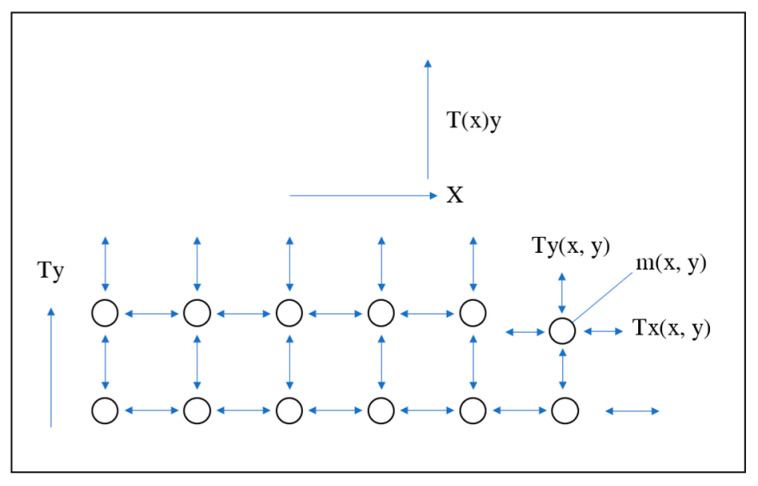
Mass and tension network for time-domain model [15].

In the time-domain model, the tire is divided into several circumferential belts from the centerline to the tread and then to the rigid bead. Then, each belt is divided into several sections and connected with the adjacent belt through transverse or circumferential tension. The shear stiffness of the tire can be considered by increasing the lateral tension. Figure 2 shows the time-domain model of the tire standing wave.

The input of the time-domain model is the radial speed of tire rolling. The radial velocity will vary according to the length of the contact patch. The belt input outside the contact patch is zero. The time-domain model [15] can be expressed as:

(2) $$F_{x}\left(x,y\right)=\left[Z\left(x+1,y\right)-Z\left(x,y\right)\right]S_{x}+\left[V\left(x+1,y\right)-V\left(x,y\right)\right]\eta_{x}$$

(3) $$F_{y}\left(x,y\right)=\left[Z\left(x,y+1\right)-Z\left(x,y\right)\right]S_{y}\left(x\right)+\left[V\left(x,y+1\right)-V\left(x,y\right)\right]\eta_{y}$$

(4) V(x,y)=V(x,y)+{[ Fx(x,y)−Fx(x−1,y)+Fy(x,y)−Fy(x,y−1)]/M(y)}dt

(5) Z(x,y)=Z(x,y)+V(x,y)dt

where $F_{x}\left(x,y\right)$, $F_{y}\left(x,y\right)$, $F_{x}\left(x-1,y\right)$ and $F_{y}\left(x,y-1\right)$ are four forces acting on the mass m(x,y). Z(x,y) is the displacement, which is obtained by multiplying the velocity acting on the mass segment by time. V(x,y) is the velocity. $S_{x}$, $S_{y}$ are the elasticity in the circumferential and transverse direction, which is obtained from the tension and length on the mass segment. $\eta_{x}$, $\eta_{y}$ are the viscous constant in the circumferential and transverse direction.

When the increment time is greater than the segment length divided by the tire speed, the segment will continue to move (the segment length in the circumferential direction). Considering the boundary condition, when *x* = 1, the speed is the speed at which the tire leaves the drum, so the reflection at the far end is the smallest. There is no force on the centerline and no displacement at the bead.

## 3. Aircraft Tire Numerical Modelling

### 3.1. 3-D Aircraft Tire Model

This paper adopts the 660 × 200 aircraft tire designed by Guilin Lanyu Aircraft Tyre Development Co., Ltd. (Guilin, China) [16,17]. The skeleton structure of a traditional bias aircraft tire has no obvious stiffness and flexibility zoning, the mechanical properties of sidewall and crown are almost the same, and the buffer layer does not play a major role. Then, the carcass of a radial tire is equivalent to pneumatic spring. The belt is equivalent to the tank track, and the “soft carcass and rigid belt” is the essence of the radial tire.

The structure of an aircraft tire is very complex. When establishing the finite element model, reasonably simplify the tire model according to the actual situation:

The tread pattern on the tire is not considered; only the longitudinal groove is considered;
The contact between rim and tire is simplified and tie constraints are set;
The drum is considered as rigid.

**Figure 3:**
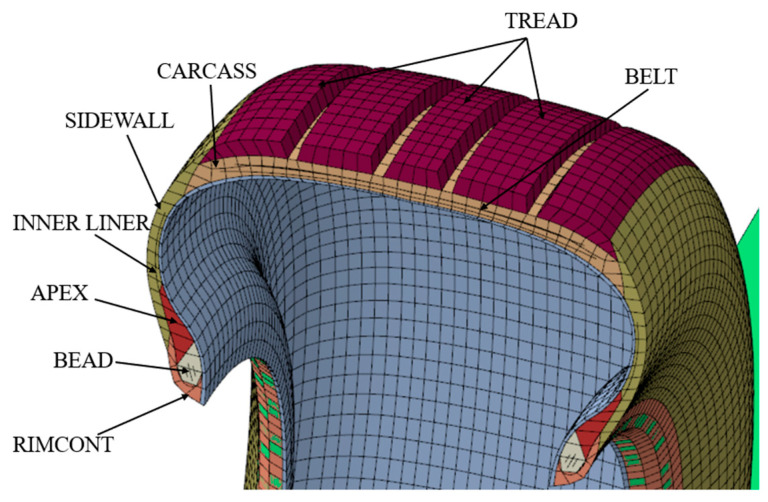
The finite element model of aircraft tire section.

In the established cross-section model, the aircraft tire studied is divided into eight components, as shown in Figure 3. Use integral reduction and hourglass control, both of which are set to the default parameter values.

The rubber material in an aircraft tire is a three-dimensional network structure composed of randomly oriented long molecular chains. It has the characteristics of low modulus and high damping and can absorb the energy brought by impact load. The nonlinear behavior of rubber is simulated by the Mooney–Rivlin constitutive equation, with a total of 22,800 C3D8R (8-node hexahedral linear reduction integral element) finite element discrete elements. The deformation potential energy of the Mooney–Rivlin model is given by the following formula [18]:

(6) $$U=\sum_{i=1}^{N}C_{i0}{\left({\bar{I}}_{1}-3\right)}^{i}+\sum_{i=1}^{N}\frac{1}{D_{i}}{\left({\bar{J}}_{e1}-1\right)}^{2i}$$

where U is the strain energy potential; ${\bar{J}}_{e1}$ is the elastic volume ratio; ${\bar{I}}_{1}$ is the first invariant of the deviatoric strain; $C_{i0}$ describes the shear behaviour of the material; and $D_{i}$ introduces compressibility. Table 1 lists the rubber material parameter values used in this work.

The properties of reinforced materials are considered to be homogeneous and orthotropic, and their parameter values are provided by the tire manufacturer. There are 22,800 C3D8R finite discrete elements in the beads. Young’s modulus, density, and Poisson’s ratio of bead core are 8.4 × 10^10^, 5900, and 0.3, respectively. The belts are defined using the surface-type SFM3D4R in the model, and Young’s modulus, density and Poisson’s ratio of steel belts are 8.4 × 10^10^, 5900, and 0.4, respectively. The reinforcement SURF_BELT-1 and SURF_BELT-2 are composed of 2600 and 2400 finite elements, respectively. A total of 10,800 finite elements are used to discretize the carcass layer. Table 2 lists the geometric properties of the reinforcement materials.

### 3.2. Dynamic Rolling Numerical Model

ABAQUS/Explicit uses the central difference method to perform explicit time integration of the equation of motion; the dynamic conditions of one incremental step are applied to calculate the dynamic conditions of the next incremental step [19]. At the beginning of the incremental step, the program solves the dynamic equilibrium equation, which is expressed as

(7) $$M\ddot{u}=P-I$$

where M is the nodal mass matrix, $\ddot{u}$ is the nodal acceleration, P is the applied external force, and I is the unit internal force;

At the beginning of the current increment step, the calculated acceleration is

(8) $${\ddot{u}}_{\left(t\right)}={\left(M\right)}^{-1}\cdot {\left(P-I\right)}_{\left(t\right)}$$

Apply the change value of speed plus the speed at the midpoint of the previous incremental step to determine the speed at the midpoint of the current incremental step:

(9) $${\dot{u}}_{\left(t+\Delta t/2\right)}={\dot{u}}_{\left(t-\Delta t/2\right)}+\frac{\Delta t_{\left(t+\Delta t/2\right)}+\Delta t_{\left(t\right)}}{2}{\ddot{u}}_{\left(t\right)}$$

The integral of velocity versus time plus the displacement at the beginning of the incremental step determines the displacement at the end of the incremental step:

(10) $$u_{\left(t+\Delta t\right)}=u_{\left(t\right)}+\Delta t_{\left(t+\Delta t\right)}{\dot{u}}_{\left(t+\Delta t/2\right)}$$

The stability limit is defined in the form of the highest frequency in the system, and the damped stability limit is defined by the following formula:

(11) $$\Delta t_{stable}=2/\omega_{\max}\left(\sqrt{1+\xi^{2}}-\xi \right)$$

where $\omega_{\max}$ is the highest frequency in the system and ξ is the critical damping at the highest frequency mode.

**Figure 4:**
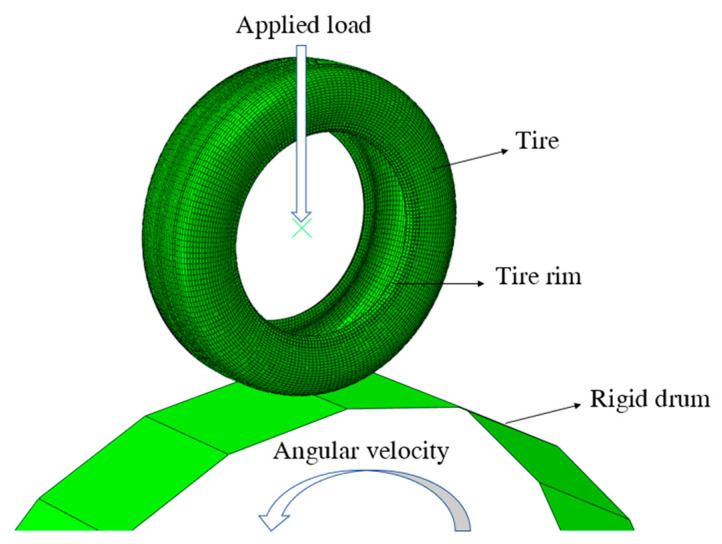
The tire-drum finite element model for standing waves.

In this paper, the Total Lagrange method in ABAQUS is used to describe the geometric nonlinearity caused by large deformation of an aircraft tire. The dynamic process of an aircraft tire accelerating from 0 to 300 km/h is simulated in ABAQUS, as shown in Figure 4. The drum model is represented by an undeformed rigid body. The contact nonlinearity between tread and drum surface is described by the penalty function method, and the friction coefficient is set to 0.5. The stiffness proportional damping of Rayleigh damping is also adopted in ABAQUS, which is set as the default value. The vertical load is applied to the center of the aviation tire, and the rotational angular velocity is applied to the center of the drum. A 16-core AMD Ryzen 93950x supercomputer (Dell, Nanjing, China) (2 CPUs, 1.6 GHz per core) carried out the simulation. The whole simulation process ran for 12 h.

## 4. Experiment and Validation

**Figure 5:**
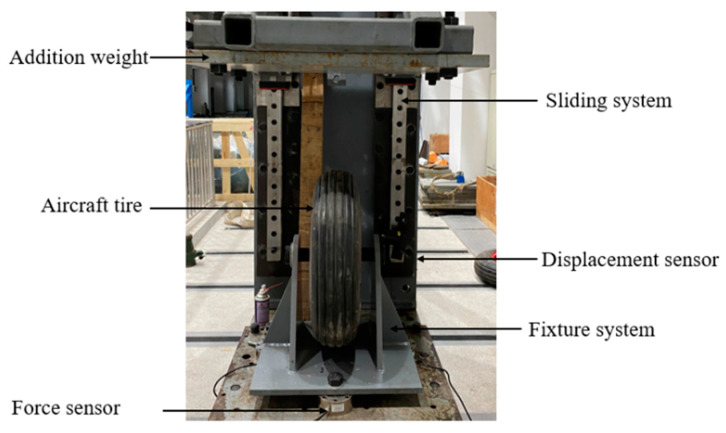
The aircraft tire experimental test.

In this section, in order to verify the proposed finite element model, the numerical simulation results are compared with the experimental data under inflation and static load. The experimental test is shown in Figure 5.

### 4.1. Inflation Scenario

**Figure 6:**
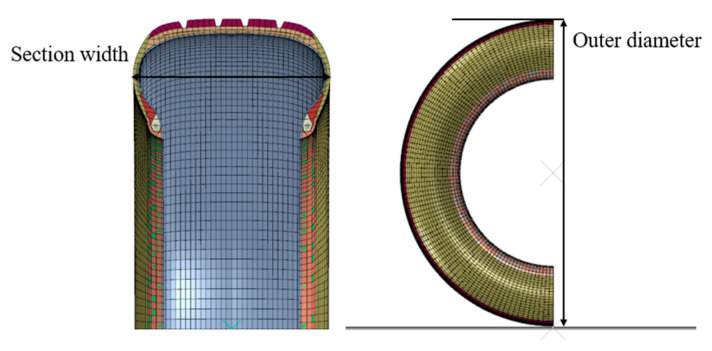
The aircraft tire cross-section under inflation.

In the case of inflation, the finite element model size under the rated inflation pressure is compared with the test tire size data in the inflation test to verify the accuracy of the finite element model, as shown in Figure 6. In order to ensure repeatability, a total of two tests were carried out. The results of simulation and test are shown in Table 3. The difference between the tire cross-section width and outer diameter is 1.7% and 0.5%. This shows the effectiveness of the two-dimensional section finite element model.

### 4.2. Static Load Scenario

**Figure 7:**
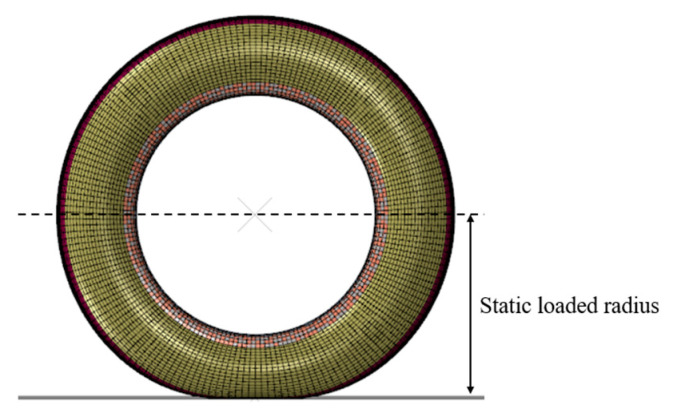
The relationship between the sinkage of aircraft tire and vertical load.

The sinkage of an aircraft tire is the radial deformation under the action of vertical load, as shown in Figure 7. FAA documents stipulate that both tire pressure and load should make the sinkage of tire in an appropriate range [20].

**Figure 8:**
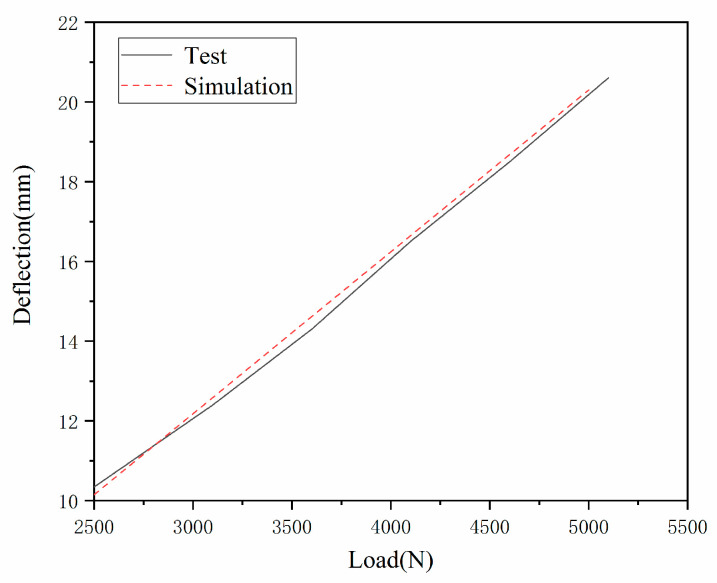
The deflection vs. vertical load.

Figure 8 shows the load deflection curve of a tire under different vertical load conditions under rated inflation pressure. The tire inflation pressure is fixed at 0.618 MPa, the loads are 5000, 4500 and 2500 N, and the simulated deflections are 20.0, 17.8, and 10.5 mm, respectively. The experimental deflections are 20.1, 18.0, 16.1, 12.0, and 10.3 mm for 5000, 4500, 4000, 3500, 3000, and 2500 N. Through the variation curve of vertical load and deflection of an aviation tire, it can be seen that the numerical simulation results are in good agreement with the experimental results and the previous research results [21].

## 5. Dynamic Simulation and Analysis during High-Speed Tire Rolling

The main purpose of this simulation is to develop a practical aircraft tire finite element model to predict the standing wave phenomenon. A 660 × 200 aircraft tire with a rigid rotating drum is used for the simulation. The simulation process accelerates from 0 to 300 km/h in 0.3 s, according to the actual situation of the aircraft landing.

**Figure 9:**
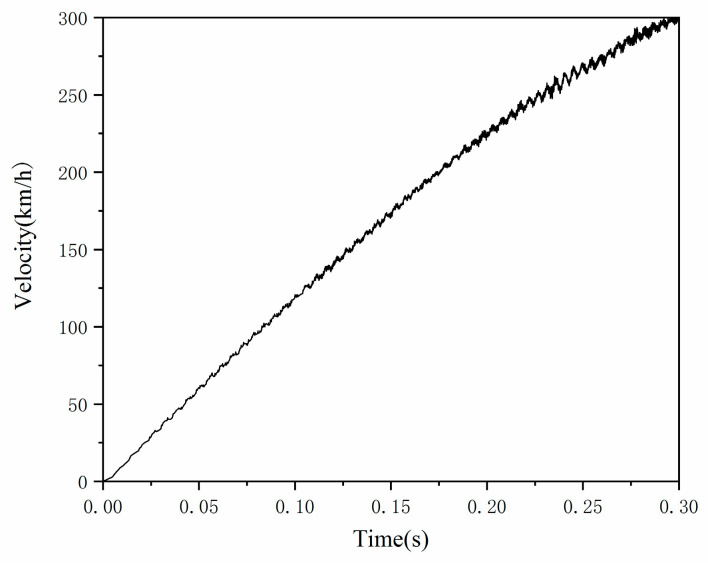
The velocities monitoring of the tire.

The numerical simulation of dynamic standing wave of aircraft tire is divided into three parts. First, different inflation pressures are applied to the inner surface of the tire, that is, standard recommended pressure 618 KPa, under-pressure 525.3 KPa (85% standard recommended pressure), and over-pressure 710.7 KPa (115% standard recommended pressure). Then, different vertical force is applied to the center of the aircraft tire, that is, standard load 10,000 N, under-load 8500 N (85% standard load), and over-load 11,500 N (115% standard N). Finally, the drum is forced to rotate clockwise from the rest position so that the tire will be gradually accelerated from 0 to 300 km/h linear velocity in 0.3 s at a constant rate, and the drum can always travel at the same speed as the tire in the whole simulation process, as shown in Figure 9.

### 5.1. Verification

**Figure 10:**
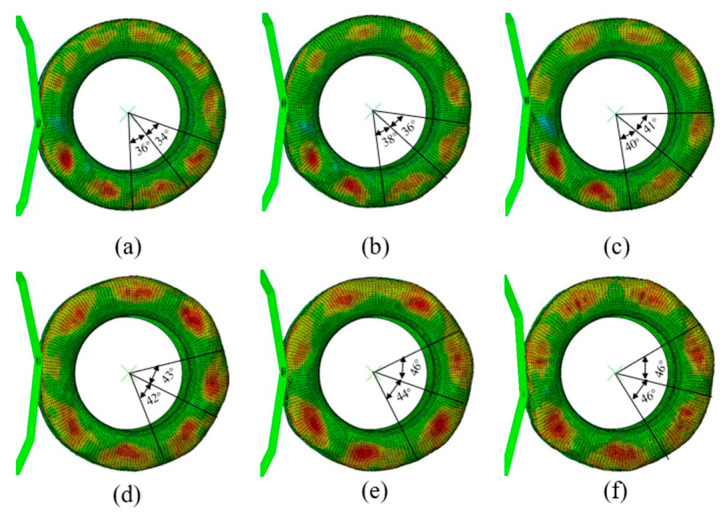
The standing wave deformation of an aircraft tire: (**a**) 200 km/h, (**b**) 220 km/h, (**c**) 240 km/h, (**d**) 260 km/h, (**e**) 280 km/h, (**f**) 300 km/h.

To verify the accuracy of the finite element model under dynamic conditions, the simulation results will be compared with the calculations based on the time-domain model. Figure 10 shows a comparison of the simulation results from 200 km/h to 300 km/h on tread standing waves within the π-arc. The closer the color is to red in the simulation graph, the greater the deformation is. The closer the color is to blue, the smaller the deformation. The angle in the curve represents the angle between two adjacent peaks over a period of time. With the gradual increase of the rolling speed, the values of the angles also increase; that is, the speed increases, the energy consumption increases, the tire deformation increases, and the sidewall wavelength increases gradually.

**Figure 11:**
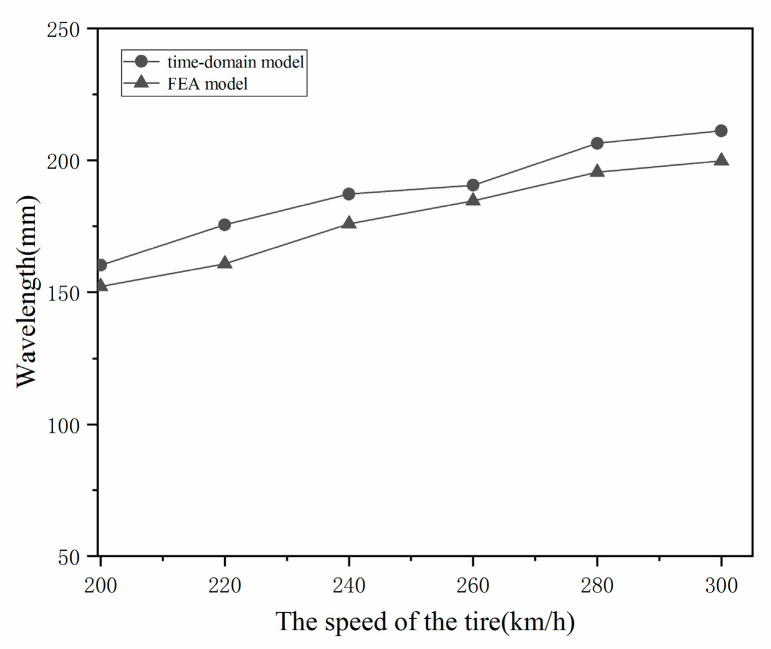
Comparison between time-domain model and FEA model results on wavelength of standing waves.

Figure 11 compares the wavelength curves calculated from the time-domain model and the FEA model. Overall, the coincidence of the two methods is accurate; that is, the wavelength gradually increases with the speed of the tire. However, the wavelength of the time-domain model is slightly longer than that of the FEA model. This difference is that the time-domain model assumes that the carcass is flat on the plane; for an actual deformed toroidal tire, the situation is not as expected. The radial distance from the axis to the crown is decreased, and the maximum cross-section is increased. The wavelength of the FEA model is smaller than that of the time-domain model, which is good proof of this.

### 5.2. Stress–Strain Field Analysis

In order to study the stress–strain deformation of the aircraft tires when standing wave phenomenon occurs, the simulation of aircraft tire at different speeds was carried out under the conditions of vertical load of 10,000 N and standard inflation pressure of 618 kPa.

**Figure 12:**
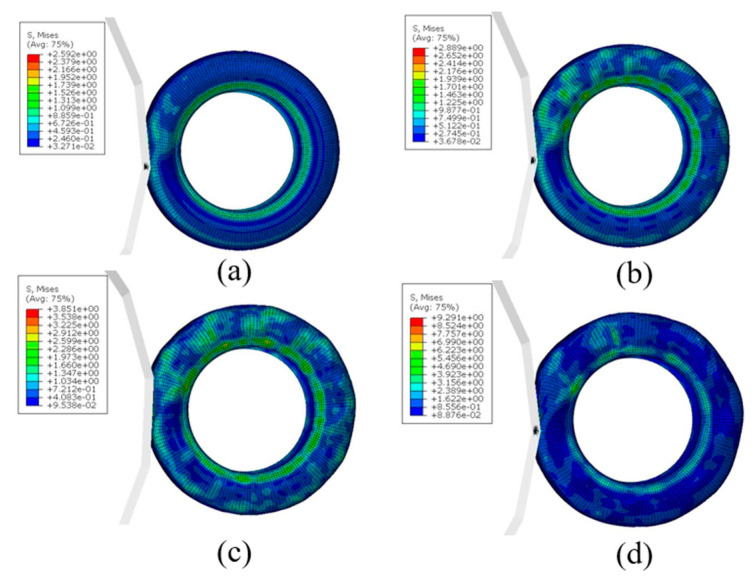
Stress field of aircraft tire at different speeds: (**a**) 130 km/h, (**b**) 150 km/h, (**c**) 170 km/h, (**d**) 190 km/h.

**Figure 13:**
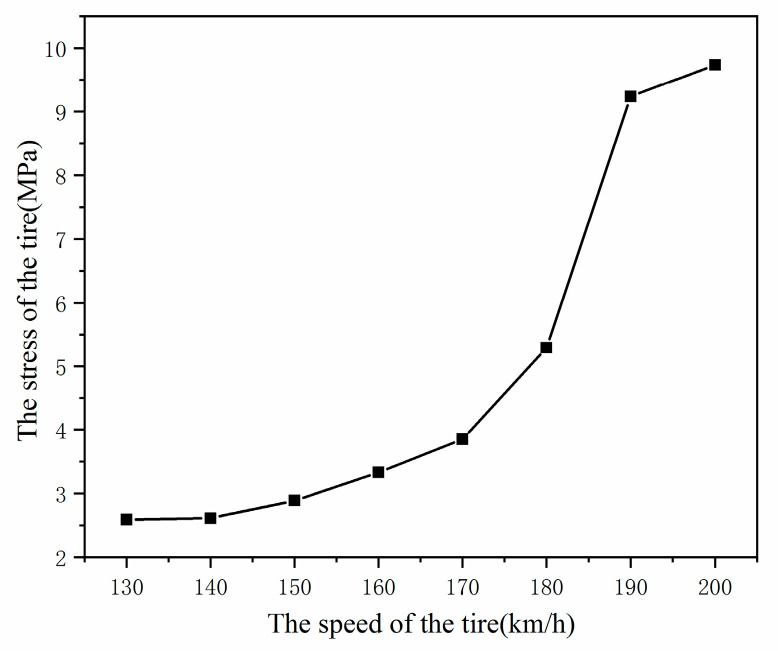
The stress changes of aircraft tire at different speeds.

Figure 12 and Figure 13 show the tire stress field when the rolling speed is V = 130 km/h, V = 150 km/h, V = 170 km/h, and V = 190 km/h. It can be seen from Figure 13 that with the increase of rolling speed, the stress level of an aircraft tire also increases. When the rolling speed of the aircraft tires reaches the critical speed V = 190 km/h, the stress level of aviation tire increases sharply from 3.85 MPa of 170 km/h to 9.29 MPa, indicating that the standing wave phenomenon has occurred at this time.

**Figure 14:**
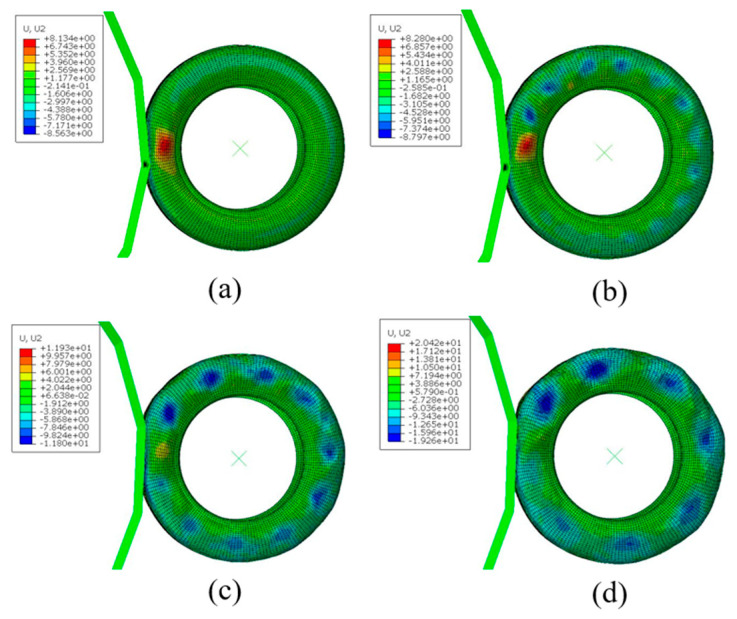
Strain field of aircraft tire at different speeds: (**a**) 130 km/h, (**b**) 150 km/h, (**c**) 170 km/h, (**d**) 190 km/h.

**Figure 15:**
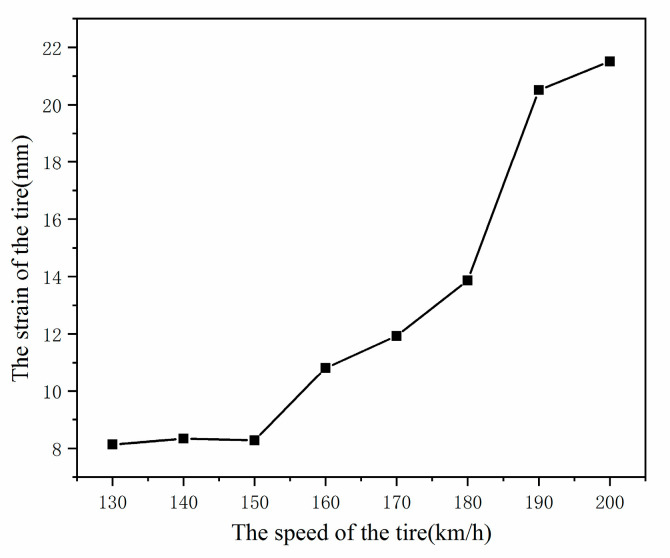
The strain changes of aircraft tire at different speeds.

Figure 14 and Figure 15 show the tire strain field when the rolling speed is V = 130 km/h, V = 150 km/h, V = 170 km/h, and V =190 km/h. It can be seen from Figure 15, when accelerating from 0 km/h to 170 km/h, that the strain level increases steadily. When the rolling speed of the aircraft tires reaches the critical speed V = 190 km/h, the strain level of an aircraft tire increases sharply from 11.9 mm of 170 km/h to 20.4 mm of 190 km/h, and the tire shape is greatly distorted, indicating that the standing wave phenomenon has occurred at this time.

### 5.3. Parametric Investigation

In this section, the effects of tire pressure and vertical load on standing wave are studied from the perspective of the internal energy of an aviation tire. Except for these two parameters, the other simulation parameters are the same as before. The simulation cases were chosen for each parameter: normal, 15% below, and 15% above, as shown in Table 4.

**Figure 16:**
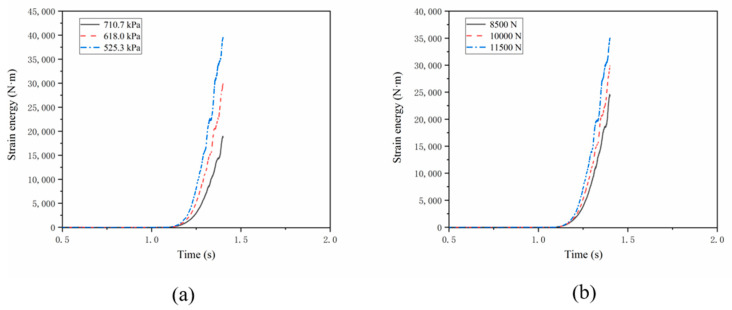
Strain energy consumption: (**a**) with fixed vertical load 10,000 N and different inflation pressure; (**b**) with fixed recommended pressure 618 kPa and different loads.

Figure 16 shows the time history of the change of tire strain energy from 0 acceleration to 300 km/h. The whole simulation process includes two steps: the inflation and the static contact step is completed in 1 s, and the transient dynamic rolling step is completed in 0.3 s. Since the drum accelerates along with the time–velocity specification (Figure 9), the tire speed at a particular time can be clearly known. It is observed that the strain energy increases significantly with the increase of tire speed. Especially when a standing wave occurs, there will be a sharp increase. As shown in Figure 16b, the strain energy increases with tire loads.

From Figure 16, we can observe the following points:

(1) With the increase of rolling speed of aircraft tire, the strain energy of aircraft tire increases significantly;
(2) After the standing wave phenomenon of aircraft tire appears, the strain energy increases sharply;
(3) The initial strain energy of an aircraft tire comes from the internal pressure of the tire. The smaller the internal pressure of the tire, the faster the strain energy curve rises when the standing wave phenomenon occurs;
(4) As shown in Figure 16a, at 0.3 s, when the rolling speed of an aircraft tire reaches 300 km/h, the strain energy consumption under the inflation pressure of 525.3 kPa is almost twice that under the inflation pressure of 710.7 kPa. The energy consumed under the standard inflation pressure of 618 kpa is one third more than that under the condition of over inflation of 710.7 kPa and one quarter less than that under the condition of inflation of 525.3 kpa.
(5) As shown in Figure 16b, when the rolling speed of aircraft tire reaches 300 km/h in 0.3 s, the greater the load of the aviation tire, the faster the strain energy curve rises when the standing wave phenomenon occurs. The energy consumed under the standard load of 10,000 n is one sixth more than that under the light load of 8500 N and one sixth less than that under the overload of 11,500 N.

The simulation results of dynamic standing waves of an aviation tire are shown in Table 5 and Table 6.

Referring to Table 5 and Table 6, the critical speed increases with the increase of inflation pressure and decreases with the increase of vertical load. This result is consistent with the theoretical and experimental results of other researchers [8,9]. Therefore, the critical speed is affected by the inflation pressure and the vertical load. The critical speed is expressed by least square fitting:

(12) $$V_{c}=-2.90925\times 10^{-4}\cdot P^{2}+0.54856\cdot P-37.7778$$

(13) $$V_{c}=-1.55556\times 10^{-6}\cdot F^{2}+0.02211\cdot F+124.4444$$

**Figure 17:**
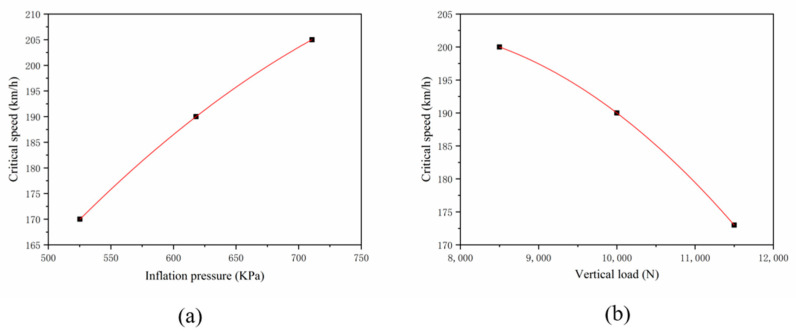
Critical speed vs. (**a**)inflation pressure and (**b**)vertical load.

As shown in Figure 17.

## 6. Conclusions

This paper established a finite element model of the aircraft tire in ABAQUS. The mechanism of standing wave generation in the process of tire rolling was analyzed by the time-domain model and FEA model and the relationship between sidewall wavelength and tire speed was studied. We compared the experimental and simulation results in the inflation scenario and static load scenario. We found that the simulation results of the finite element model are in good agreement with the experimental results. According to the actual situation of the aircraft landing, the FEA model successfully predicted and visualized the standing waves phenomenon. The simulation results show that tire strain energy increases significantly with the increase of tire speed. Especially when a standing wave occurs, there will be a sharp increase. The parameter investigation shows the critical speed is proportional to the square of the tire inflation pressure but decreases with the vertical load.

Based on the ABAQUS finite model, the conclusions are as follows:

(1) The formation of standing waves leads to an increase in strain energy consumption of the aircraft tire;
(2) When the inflation pressure is lower than the manufacturer’s recommended value, the critical speed at which the standing wave starts will decrease;
(3) When the load of the aircraft tire increases, the critical speed of the standing wave will decrease and the tire deformation will be more obvious;
(4) These conclusions can help aircraft tire designers and manufacturers gain a better understanding of this critical phenomenon, and they can improve the safety of aircraft landing to a certain extent.

## Figures and Tables

**Table 1:** Rubber material parameter values.

Material	C10	C01	D1	Density (kg/m³)
Tread	1.0000	0.0000	0.0198	1100
Carcass	1.1390	0.0000	0.0240	1100
Sidewall	1.0000	0.0000	0.0600	1100
Inner liner	0.9530	0.0000	0.0300	1100
Apex	1.6060	0.0000	0.0200	1100
Rimcont	1.4060	0.0000	0.0200	1100
Bead	0.6710	0.0000	0.0300	1100

**Table 2:** The geometric parameter values of reinforcement material.

Surface	Cross-Sectional (mm^2^)	Spacing (mm)	Orientation (Degree)
SURF_BELT-1	0.3150	1.2200	66
SURF_BELT-2	0.3318	1.2700	114
BEAD	0.1869	1.0100	90
PLY	0.3848	1.2700	0

**Table 3:** Tire inflation test and simulation results.

Test Object	Test	Simulation	Differnce
Section width (mm)	182.3	185.5	1.7%
Outer diameter (mm)	651.2	654.4	0.5%

**Table 4:** Parameter conditions adopted in the simulation.

Variable	Units	Low	Normal	High
Pressure	kPa	525.3	618	710.7
Vertical load	N	8500	10,000	11,500

**Table 5:** Standing wave simulation results based on inflation pressure change.

Pressure (kPa)	Critical Speed (km/h)
525.3	170
618	190
710.7	206

**Table 6:** Standing wave simulation results based on vertical load change.

Vertical Load (N)	Critical Speed (km/h)
8500	200
10,000	190
11,500	173

## Data Availability

Not applicable.
